# Optimization of Detection Accuracy of Closed-Loop Optical Voltage Sensors Based on Pockels Effect

**DOI:** 10.3390/s17081723

**Published:** 2017-07-27

**Authors:** Wei Deng, Hui Li, Chunxi Zhang, Pengjie Wang

**Affiliations:** School of Instrumentation Science and Opto-electronics Engineering, Beihang University, Beijing 100191, China; deng1517101@buaa.edu.cn (W.D.); zhangchunxi@buaa.edu.cn (C.Z.); wpjzy1617130@buaa.edu.cn (P.W.)

**Keywords:** optical voltage sensor, detection accuracy, closed-loop detection

## Abstract

The influence of optical parameters on the performance of closed-loop optical voltage sensors (OVSs) based on Pockels effect is analyzed and a control algorithm is proposed to suppress the nonlinearity caused by the unideal parameters of optical devices for optimizing the detection precision of OVSs. First, a quantified model of the feedback phase demonstrates how the optical parameters of optical devices (including light source, polarizer, 45° fusion point, Faraday rotator and half-wave plate) result in the nonlinearity of closed-loop OVSs. Then, the parameter indexes of different optical devices are put forward to instruct the manufacturing process of the optical system. Furthermore, a closed-loop control algorithm is investigated to improve the measurement accuracy of nonlinear OVSs considering the unideal parameters. The experiment results indicate that additional bias caused by undesirable optical parameters is obviously decreased so that the measurement accuracy of OVSs satisfies the demand of IEC60044-3 for 0.1 level measurement accuracy, which verifies the effectiveness and correctness of the methods for suppressing the impact of unideal optical parameters on OVSs.

## 1. Introduction

Optical voltage sensors (OVSs) are receiving increased attention as a key component of power systems, owing to the advantages of their small size, light weight, high bandwidth, safety, intrinsic immunity to electro-magnetic interference and so on [1,2]. In particular, OVSs based on Pockels effect have attracted a great number of researchers due to their currently wide applications in electric power industry [3,4].

Over the past decade, much research has been devoted to optimizing optical parameters and signal processing methods for improving the detection precision of OVSs based on Pockels effect. Lee proposed a compensation method utilizing double light paths which can counteract the effects of both unwanted linear and circular birefringences on the OVS [5], and the method of annealing crystal was then investigated to decrease the temperature-dependent birefringences [6]. Franjo Cecelia et al. designed an optimal optical alignment of the crystal to compensate for the temperature error of the quarter-wave plate for enhancing the sensitivity of OVSs [7]. A quasi-reciprocal reflective optical circuit adopting the Faraday rotator was presented to promote the reciprocity of polarized light in the transmission path of OVSs [8], and the voltage sensing scheme with dual-crystal structure was proposed to eliminate the additional birefringence of Bi_4_Ge_3_O_12_ (BGO) crystal for guaranteeing the reciprocity of the voltage sensing process [9]. Then, the optimization methods of a reciprocal dual-crystal sensing system were researched for optimizing the temperature performance of OVSs in an engineering environment [10]. Xia et al. introduced the thermodynamic model including an electric field, stress and temperature field, and analyzed the impact of optical properties such as temperature and sulfur hexafluoride (SF6) for suppressing the environmental factors [11]. Chu et al. utilized a polymeric integrated optic system consisting of coupler, polarizer, phase modulator and polarization converter to enhance the anti-disturbance ability of OVSs [12].

Signal processing methods are also of great importance in optimizing the measurement accuracy of OVSs. A digital signal processor using the arcsine function was designed to overcome the defect of optical nonlinearity for high voltage applications [13]. Feng et al. proposed a detection circuit to acquire the AC and DC components of the sensing signals from two light paths for improving the linearity and accuracy performance of AC voltage measurements [14,15]. However, in the above open-loop signal processing scheme it is difficult to suppress the drift of optical parameters of OVSs. A closed-loop control algorithm considering the fluctuation of interference intensity was put forward to guarantee the exponential stability of OVSs [16]. Furthermore, a detection method based on four-state modulation technology was investigated to demodulate and control the gain drift of the phase modulator [17]. However, the above parameters optimized by closed-loop control methods are only the intensity of interference light or gain of the phase modulator. The quantified relationship between all key optical parameters and the performance of closed-loop OVSs is difficult to obtain, precisely due to interaction of multi-optical parameters. Furthermore, the closed-loop control method presents a great challenge to eliminate the impact of unideal optical parameters on OVS accuracy.

In this paper, we firstly investigate the quantified relationship between the parameters of all optical devices and feedback phase of closed-loop OVSs utilizing the dual-crystal sensing method. Then, the requirements of the optical parameters are analyzed to satisfy the demand of IEC60044-3 for the 0.1 level measurement error, so that we obtain a certain range of each optical parameter, limited in the manufacturing process. Considering the fluctuation of optical parameters, a control algorithm is further proposed to improve the measurement accuracy of OVSs.

## 2. The Model of Feedback Phase of Closed-Loop OVS Considering Unideal Optical Parameters

**Figure 1:**
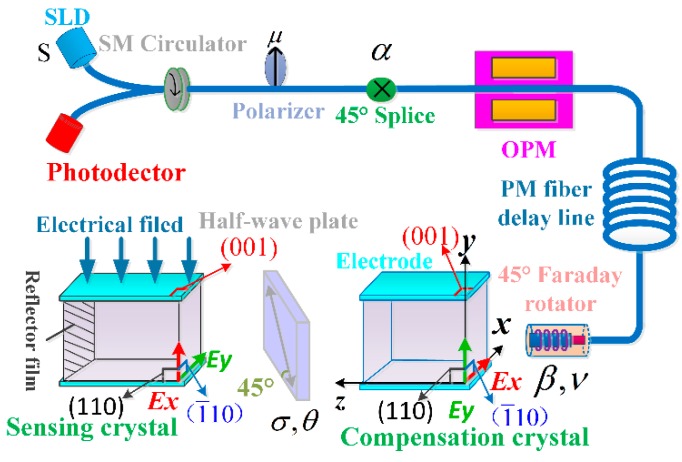
Configuration of optical voltage sensors (OVS) with dual-crystal scheme.

The work principle of the proposed dual-crystal OVSs based on Pockels effect is shown in Figure 1. The 45° splice makes the light emitted by the light source become two orthogonal linear-polarized lights with equal energy. The two orthogonal polarized lights pass through the optical phase modulator (OPM) to the fiber delay line. The vibration direction of the linear-polarized lights is rotated 45° by the Faraday collimation rotator, which realizes the reciprocity of two orthogonal polarized lights in the polarization-maintaining (PM) fiber delay line [8]. Then the applied voltage generates Pockels phase between the two orthogonal linear-polarized lights, only in the sensing crystal. The two BGO crystals are both in the transverse configuration, and the axes (x, y and z) indicated in Figure 1 are the new axes induced by the electric field. The half-wave plate may compensate for the thermal-stress birefringence error of the BGO crystal by exchanging the vibration modes of two polarized lights in the dual-crystal structure [9]. The scheme of the optical system may optimize the anti-disturbance ability of OVSs in engineering practice. However, the complex influence of optical parameters on OVSs is introduced, which results in the difficulty to optimize the detection sensitivity of OVSs.

In addition, the quantified relationship between optical parameters and the feedback phase of OVSs is not clear. The key optical parameters of a dual-crystal system include *S*, *μ*, *α*, *β*, *σ*, *θ* and *ν*, where *S* is the polarization degree of light sources, *μ* is the extinction coefficient of the polarizer, *α* is the actual angle of 45° fusion point, *ν* is the rotation angle of Faraday rotator, *β* is the axis-angle between the Faraday rotator and BGO crystal, *θ* is the phase delay angle of half-wave plate and *σ* is the axis-angle between BGO crystal and half-wave plate.

**Figure 2:**
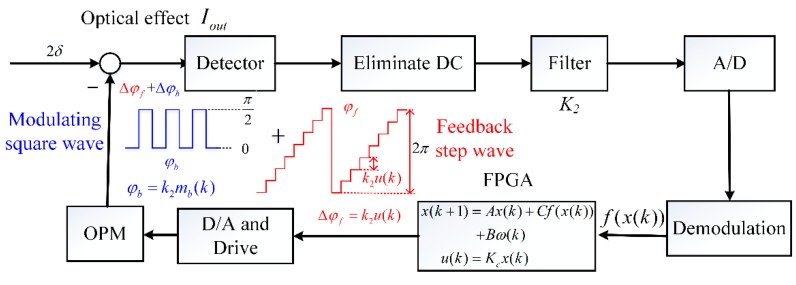
The detection scheme of closed-loop OVS.

The closed-loop scheme of OVSs based on square wave modulation is shown in Figure 2. The square wave modulation *ϕ_b_*(*t*) introduces a modulation phase Δ*ϕ_b_*(*t*) with the amplitude ±π/2, which changes the function of the interference light intensity about Pockels phase from cosine to sine. *Τ* = 3.6 μs is the transit time between the light passing and retracing the OPM. The feedback phase of closed-loop OVS generated by step wave compensates for the Pockels phase shift, which makes OVSs work at null point of the sine function of the interference light intensity. The modulation and feedback phase technology guarantees the OVS may achieve ideal dynamic work range, linearity and sensitivity.

The modulation and feedback step wave are generated in the Field Programmable Gate Array (FPGA) by digital signal. The feedback step is the feedback control *u*(*k*), and the maximum amplitude of digital step wave is designed as 2^15^. The expression of the digital modulation signal is as follows

(1) $$m_{b}(k)=\{\begin{array}{cc} \frac{2^{15}}{4} & 2k\tau <t<(2k+1)\tau \\ 0 & (2k+1)\tau <t<2(k+1)\tau \end{array},\;(k=0,1,2\ldots \infty )$$

Then, the digital modulation signal and feedback step wave are respectively converted into *ϕ_b_*(*t*) and *ϕ_f_*(*t*) through the D/A drive and OPM, and the gain of conversion is *k*_2_ = 2π/2^15^. Thus, *ϕ_b_*(*t*) can be acquired by

(2) $$\varphi_{b}(t)=\{\begin{array}{cc} \frac{\pi }{2} & 2k\tau <t<k+1)\tau \\ 0 & (2k+1)\tau <t<2(k+1)\tau \end{array},\;(k=0,1,2\ldots \infty )$$

The interference intensity detected by photodetector can be expressed as

(3) $$D(t)=\frac{1}{2}k_{1}K_{2}I_{0}\left[1+\cos\left(2\delta -\varphi_{b}(t)+\varphi_{b}(t-\tau )-\varphi_{f}(t)+\varphi_{f}(t-\tau )\right)\right]$$

where *I*_0_ is about 600 μW which is the intensity of light source, *k*_1_ is the optical loss and *K*_2_ is the gain of the detector and *2δ* is the Pockels phase. When the light passes and retraces to OPM, the feedback signals are *ϕ_f_*(*t* − *τ*) and *ϕ_f_*(*t*) and the modulation signals are *ϕ_b_*(*t* − *τ*) and *ϕ_b_*(*t*), respectively. Thus, we obtain the total feedback phase Δ*ϕ_f_*(*t*) = *ϕ_f_*(*t*) − *ϕ_f_*(*t* − *τ*) = *k*_2_*μ*(*k*) and the total modulation phase Δ*ϕ_b_*(*t*) = *ϕ_b_*(*t*) − *ϕ_b_*(*t* − *τ*). So, Equation (3) can be equivalent to

(4) $$D(t)=\frac{1}{2}k_{1}K_{2}I_{0}\left[1+f_{b}(t)\cdot \sin\left(2\delta -\Delta \varphi_{f}(t)\right)\right]$$

where *f_b_*(*t*) is the square wave with amplitude ±1 and period 2*τ*, and *f_b_*(*t*) is caused by the modulating phase Δ*ϕ_b_*(*t*). We can see that the signal of a closed-loop error is modulated to the frequency around 1/(2*τ*). Thus, we design the band-pass filter to eliminate the DC component and high frequency disturbances. Then, we obtain the result of demodulation [*k*_1_*K*_2_*I*_0_*sin*(2*δ* − Δ*ϕ_f_*(*t*))]/2 in the digital circuit by FPGA. The filter not only eliminates the DC component and the high-frequency disturbances, but also amplifies the closed-loop error. The gain of the filter is 15. A crystal frequency of around 40 MHz is input into the FPGA to divide modulation and demodulation timing for achieving synchronization, which is not required to be reset from time to time.

The polarization degree of light source *S*, the extinction coefficient of polarizer *μ* and the actual angle of the 45° fusion point *α* are the parameters before the two polarized lights with equal energy are generated. Considering the principle of modulation and closed-loop control, we firstly analyze the impact of *S*, *μ* and *α* on the feedback phase of OVS. Then, we can obtain the intensity of interference light *I_out_* written as

(5) $$\begin{array}{cc} I_{out} & =\frac{I_{0}}{16}[4((1+S)+\mu^{4}(1-S)+2\mu^{2})-2(1+\cos4\alpha )((1+S)+\mu^{4}(1-S)+2\mu^{2})-\\ & 2(1-\cos4\alpha )((1+S)+\mu^{4}(1-S)-2\mu^{2})\cos(2\delta -\varphi (t)+\varphi (t-\tau ))] \end{array}$$

where *I_out_* is the intensity of interference light before detector, *ϕ*(*t*) is the changed phase of polarized light and is obtained by *ϕ(t)* = *ϕ_b_(t) + ϕ_f_(t)* when the light passes through the OPM. In the closed-loop OVS, the demodulated results are only related with the AC terms of the interference intensity due to the DC being eliminated by the circuit design, as shown in Figure 2. Because of the method of closed-loop OVSs, the OVS demodulates the closed-loop error from the AC component of the interference light intensity. The closed-loop control makes Δ*ϕ_f_*(*t*) = 2*δ*, so that the closed-loop error Δ*ϕ* always work at zero point where Δ*ϕ* = 2*δ* − Δ*ϕ_f_*(*t*). The result of demodulation is [*k*_1_*K*_2_*I*_0_*sin*(2*δ* − Δ*ϕ_f_*(*t*))]/2. Thus, the change of *I*_0_ due to environmental perturbations can be ignored. In addition, the result of modulation is [*k*_1_*K*_2_*I*_0_*f_b_*(t)*sin*(2*δ* − Δ*ϕ_f_*(*t*))]/2, which means that the closed-loop error is modulated to 1/(2*τ*) frequency and will not be eliminated by the filter. The modulated phase 2δ can be DC phase or AC phase through the design of the bandwidth of the band-pass filter shown in Figure 2, where the Pockels phase is given by 2*δ* = 2π*γ*_41_*n*_0_^3^*Ul*/*λ*_0_*d*, *γ*_41_ is the electro-optical coefficient, *n*_0_ is the refractive index of the BGO crystal, *λ*_0_ is the wavelength of light, *l* is the length of crystal along the direction of light propagation, *d* is the thickness of crystal along the direction of the applied voltage, and *U* is the applied voltage. Thus, the OVS can realize AC/DC voltage detection. The DC component of the interference signal is eliminated by a filter circuit, which does not participate in the process of demodulation. Therefore, we only need to analyze the modulated signal in Equation (5)

(6) $$I_{out-AC}=-\frac{I_{0}}{8}(1-\cos4\alpha )[(1+S)+\mu^{4}(1-S)-2\mu^{2}]\cos(2\delta -\varphi (t)+\varphi (t-\tau ))$$

The ideal value of *I*_out-AC_ is −*I*_0_[cos(2*δ* − *ϕ*(*t*) + *ϕ*(*t* − *τ*))]/2. So, the normalized amplitude (*NA*) of the modulated signal is expressed as

(7) $$NA=\frac{I_{out-AC}}{I_{out-AC-ideal}}=\frac{1}{4}(1-\cos4\alpha )[(1+S)+\mu^{4}(1-S)-2\mu^{2}]$$

**Figure 3:**
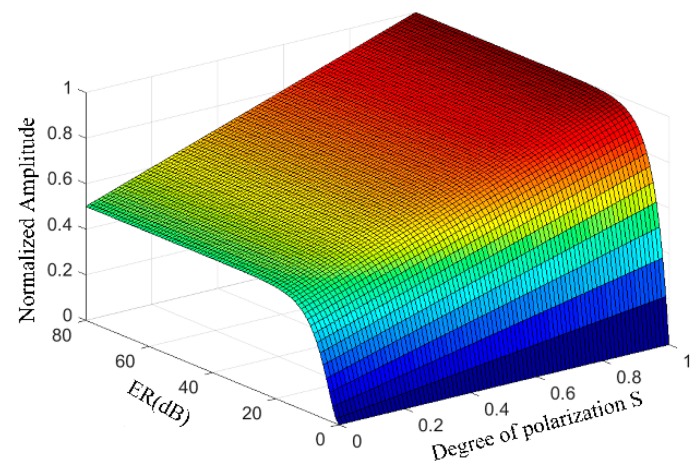
The influence of polarizer degree (*S*) and extinction ratio (*ER*) on the normalized amplitude of the AC component where *ER* = −10 lg *μ*^2^.

Then, we simulate the influence of polarization degree *S*, extinction ratio (*ER*) and axis angle *α* on *NA* by Equation (7). We can see the parameters *S*, *μ* and *α* before the incident of light splitting into orthogonal linearly polarized lights, but the light intensity contains Pockels phase due to the interference of two orthogonal polarized lights. The angle *α* is controlled within 45 ± 1° in the manufacturing process. Based on the Equation (7), we find that the parameters *α*, *S* and *μ* only affect the intensity of interference light, not feedback phase. Therefore, none of the parameters *S*, *μ* or *α* is the main factor that affects the detection accuracy of closed-loop OVSs. However, the signal-noise ratio of the closed-loop error Δ*ϕ* will be affected if the *NA* is too small. Based on simulation results shown in Figure 3, we obtain that the extinction ratio *ER* should be greater than 30 dB and polarizer degree (*S*) should be greater than 0.6, respectively. Thus, the normalized amplitude may keep no less than 80% of the ideal maximum.

The half-wave plate is the key optical component to achieve the reciprocity of the voltage-sensitive element. Next, we analyze the influence of an unideal phase delay angle of the half-wave plate on the feedback phase, considering square-wave modulation and step wave feedback. The intensity of interference light can be obtained as follows

(8) $$\begin{array}{cc} I_{out} & =|E_{out}^{*}\cdot E_{out}|=\frac{I_{0}}{2}\left\{\begin{array}{l} 1-\cos\left[\varphi (t)-\varphi (t-\tau )-2\delta \right]+\frac{\left(1+\cos2\delta \right)\left(1-\cos4\theta \right)}{4}\cdot \cos\left[\varphi (t)-\varphi (t-\tau )\right]\\ +2\sin2\delta \cdot \cos^{2}\frac{\theta }{2}\cdot \sin\left[\varphi (t)-\varphi (t-\tau )\right] \end{array}\right\}\\ & =\frac{I_{0}}{2}\left\{1+[\frac{\left(1+\cos2\delta \right)\left(1-\cos4\theta \right)}{4}-\cos2\delta ]\cdot \cos\left[\varphi (t)-\varphi (t-\tau )\right]+(2\cos^{2}\frac{\theta }{2}-1)\sin2\delta \cdot \sin\left[\varphi (t)-\varphi (t-\tau )\right]\right\} \end{array}$$

where *E_out_* is the total transmission matrix of the light from the light source to the detector.

Digital closed-loop detection with square-wave phase modulation and step-wave feedback is utilized to lock the interference light intensity in the null point of sine function and demodulate Pockels phase. Thus, Equation (8) can be simplified as

(9) $$\begin{array}{cc} I_{out} & =\frac{I_{0}}{2}\left\{\begin{array}{l} 1+[\frac{\left(1+\cos2\delta \right)\left(1-\cos4\theta \right)}{4}-\cos2\delta ]\cdot \cos(\varphi_{b}+\varphi_{f})\\ +(2\cos^{2}\frac{\theta }{2}-1)\sin2\delta \cdot \sin(\varphi_{b}+\varphi_{f}) \end{array}\right\}\\ & =\frac{I_{0}}{2}\left[1\mp \sin\left(\Delta \varphi_{f}(t)-f(2\delta )\right)\right] \end{array}$$

where *f(2δ)* is a function about the Pockels phase 2*δ* and the angle *θ*. The feedback phase Δ*ϕ_f_*(*t*) is generally designed to counteract the Pockels phase, which means the AC term of interferometer output is equal to zero. Thus, we obtain that Δ*ϕ_f_*(*t*) − *f(2δ)* ≈ 0. So the feedback phase Δ*ϕ_f_*(*t*) can be written as

(10) $$\begin{array}{cc} \Delta \varphi_{f}(t) & =f(2\delta )=actg\frac{-4\cos\theta \sin2\delta }{\left(3+\cos4\theta \right)\cos2\delta -(1-\cos4\theta )}\\ & \approx {\frac{-4\cos\theta \cos2\delta [\left(3+\cos4\theta \right)\cos2\delta -(1-\cos4\theta )]+4\left(3+\cos4\theta \right)\cos\theta \sin^{2}2\delta }{{\left[\left(3+\cos4\theta \right)\cos2\delta -(1-\cos4\theta )\right]}^{2}+{\left(-4\cos\theta \sin2\delta \right)}^{2}}|}_{2\delta =0}2\delta \\ & =-\frac{2\cos\theta }{\left(1+\cos4\theta \right)}2\delta \end{array}$$

**Figure 4:**
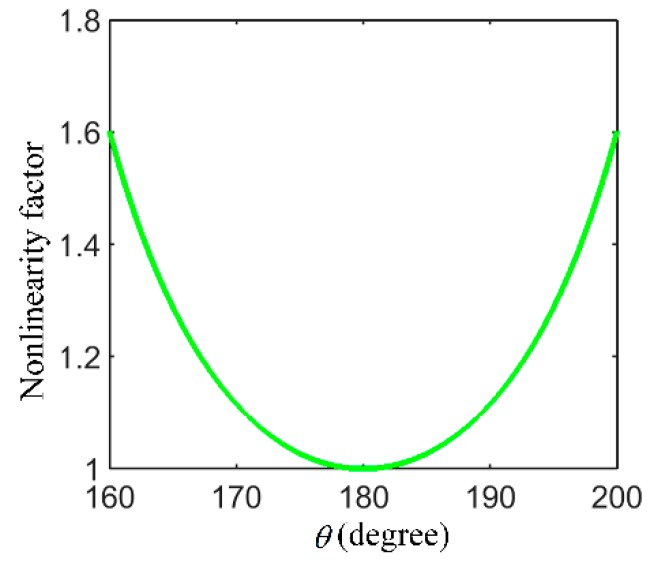
The nonlinearity factor of the OVS system with *θ* varying 160 ~ 200°.

We can see the nonlinearity factor (*NF*) of the feedback phase is *NF* = −2cos*θ*/(1 + cos4*θ*), where the angle *θ* is 180 degree in ideal conditions. In addition, the *NF* of OVSs changes with the delay angle *θ*, which demonstrates the unideal optical parameters leading to the nonlinearity of OVS as shown in Figure 4.

Furthermore, we analyze the effect of unideal multi-optical parameters on closed-loop OVS system considering parameters *β*, *σ*, *ν* and *θ*. Using the similar proof of the Equations (8)–(10), the feedback phase of closed-loop OVS Δ*ϕ_f_*(*t*) can be represented by

(11) $$\Delta \varphi_{f}(t)=actg\frac{-4\cos\theta \sin^{2}2\sigma \cdot \sin2\nu \cdot \sin2\beta \cdot \sin2\delta }{\left(\cos4\theta -\cos4\nu -\cos4\beta +\cos8\sigma \right)\cos2\delta -\left(2+\cos4\nu -\cos4\beta -\cos8\sigma -\cos4\theta \right)}$$

**Figure 5:**
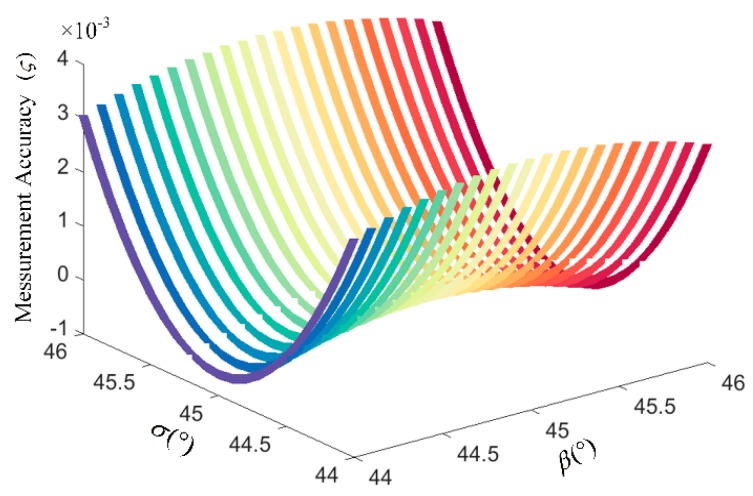
The influence of *β* and *σ* on the measurement accuracy of closed-loop OVSs.

According to the Equation (11), we firstly analyze the impact of axis angles *β* and *σ* on OVS measurement accuracy, as shown in Figure 5. The relative error is calculated by Equation *ς* = (Δ*ϕ_f_*(*t*) − 2*δ*)/2*δ*. In the modern manufacturing process, the error of the axial angles *β* and *σ* can be controlled within ±0.1° by fiber fusion splicer. Thus, the simulation results indicate that the impact of *β* and *σ* on the measurement accuracy of OVSs will not be greater than 0.01%, which is much better than the demand of IEC60044-3 for the 0.1 level measurement accuracy, as shown in Figure 5.

However, the rotation angle *ν* and the delay angle *θ* belong to the intrinsic parameters of optical devices, which may change with temperature or other environmental factors. Considering the parameters *ν* and *θ*, the feedback phase of the closed-loop OVS can be rewritten as follows

(12) $$\Delta \varphi_{f}(t)=actg\frac{-4\cos\theta \cdot \sin2\nu \cdot \sin2\delta }{\left(2+\cos4\theta -\cos4\nu \right)\cos2\delta -\left(2+\cos4\nu -\cos4\theta \right)}$$

**Figure 6:**
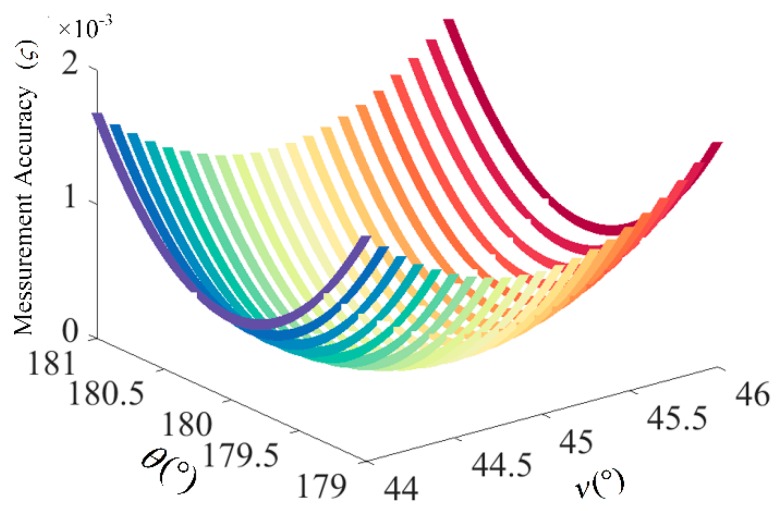
The influence of *ν* and *θ* on the measurement accuracy of closed-loop OVS.

The simulation results for the influence of *ν* and *θ* on the measurement accuracy of OVSs are shown in Figure 6. We can see that if the error of *θ* is more than 0.9° or the error of *ν* is more than 1.2°, the measurement accuracy will be greater than 0.1%, which exceeds the requirements of IEC60044-3 for 0.1 level measurement accuracy. Besides, the measurement error due to the interaction between the deviation of *ν* and *θ* is greater than the error of any angle. The unideal phase delay angle of half-wave plate *θ* and rotation angle of Faraday rotator *ν* cause the difficulty in improving the detection accuracy of OVSs, so that the closed-loop control algorithm is very important in suppressing the fluctuation of optical parameters for improving the measurement accuracy of OVSs.

## 3. Suppression Method

We consider the unideal parameters *ν* and *θ* resulting in the nonlinearity of OVS, then the dynamic equations of the closed-loop system are written as

(13) $$\begin{array}{lll} x(k+1) & = & Ax(k)+Cf(x(k))+B\omega (k)\\ u(k) & = & K_{c}x(k) \end{array}$$

where, $A=\left[\begin{array}{cccc} 1 & 1 & 0 & \cdots \\ 0 & 1 & 1 & 0\\ \cdots & \cdots & \cdots & 1\\ 0 & \cdots & 0 & 1 \end{array}\right]$, $B=\left[\begin{array}{c} 0\\ 0\\ \cdots \\ \frac{I_{0}}{2}k_{1} \end{array}\right]$, $C=\left[\begin{array}{c} 0\\ 0\\ \cdots \\ \frac{I_{0}}{2}k_{1} \end{array}\right]$, $x(k)\in R^{n}$ is the state variable with the initial condition $x(k_{0})$, $\omega (k)\in L_{2}(0,\infty )$ describes the optical noise and $K_{c}\in R^{1\times n}$ is the feedback gain matrix of the control and *u*(*k*) is both the feedback control and the output of the OVS.

Considering optical noise, we propose a closed-loop control algorithm with an exponential *H*_∞_ performance to suppress the nonlinearity introduced by undesirable optical parameters for improving the detection accuracy of OVSs. Before presenting the main results, a definition as below is needed.

**Definition** **1.** *[18,19]: For given scalars γ > 0, system (13) is said to be exponentially stable in the mean-square sense with an exponential H_∞_ performance γ, if it is exponentially stable and there holds* $\frac{1}{n}\sum_{s=k_{0}}^{\infty }E\{e^{T}(s)e(s)\}\leq \gamma^{2}\sum_{s=k_{0}}^{\infty }\omega^{T}(s)\omega (s)$ *for all non-zero* $\omega (k)\in L_{2}(0,\infty )$ *under zero initial conditions*.

For suppressing the effect of unideal optical parameters and optical noise on the detection accuracy of OVS, a sufficient *H*_∞_ performance condition for system (13) is presented in the following theorem, which guarantees the nonlinear OVS (13) is exponentially stable with the initial condition $x(k_{0})$.

**Theorem** **1.** *For a given scalar 0 < λ < 1, the nonlinear system* (8) *solves the mean-square exponential stability problem with a H_∞_ performance index γ, if there exist positive definite matrices* $P\in R^{n\times n}$*, a positive constant ε and the feedback gain matrix* $K_{c}\in R^{1\times n}$*, such that*

(14) $$\Theta =\left[\begin{array}{cccc} -\lambda^{2}P+\frac{1}{n}I & \varepsilon k_{l}k_{2}K_{c}G & 0 & \Omega^{T}P\\ {}* & -\varepsilon I & 0 & 0\\ {}* & * & -\gamma^{2}I & 0\\ {}* & * & * & -P \end{array}\right]<0$$

*where, I, P, G are the identity matrix with appropriate dimension and 0 is the zero matrix with appropriate dimension, Ω* = *[A C B]*.

**Proof.**  See the Appendix A.

Considering the closed-loop system with parameter uncertainty, we assume that the variation of the optical parameters are $\theta \in [175^{{}^{\circ}}\;185^{{}^{\circ}}]$, $\nu \in [40^{{}^{\circ}}\;50^{{}^{\circ}}]$, $\beta \in [44.9^{{}^{\circ}}\;45.1^{{}^{\circ}}]$, $\sigma \in [44.9^{{}^{\circ}}\;45.1^{{}^{\circ}}]$. Thus, *k_l_* = 1.06 is the maximum non-linearity factor due to the mainly unideal optical parameters *ν* and *θ*. The other parameters are given as follows: 0< *λ* < 1, *ε* > 0, *γ* is the *H*_∞_ disturbance attenuation level and we set *γ* = 0.2, *n* = 2, $A=\left[\begin{array}{cc} 1 & 1\\ 0 & 1 \end{array}\right]$, $B={\left[\begin{array}{cc} 0 & \frac{I_{0}}{2}k_{1} \end{array}\right]}^{T}$, $C={\left[\begin{array}{cc} 0 & \frac{I_{0}}{2}k_{1} \end{array}\right]}^{T}$, $P=G=I=\left[\begin{array}{cc} 1 & 0\\ 0 & 1 \end{array}\right]$. Then the feedback gain matrix *K_c_* = [2.445 1.01] can be obtained according to Theorem 1.

In this work, we investigate the quantified model of the feedback phase of a closed-loop OVS considering unideal optical parameters. According to the analysis results, *S*, *μ* and *α* only affect the intensity of interference light, instead of the accuracy of closed-loop OVSs. Unideal parameters including *β*, *σ*, *ν* and *θ* lead to the nonlinearity of OVSs, but we can limit the axis angles *β* and *σ* to less than ±0.1° in the manufacturing process of OVSs by fiber fusion splicer. So we can see that the impact of *ν* and *θ* is the main contributor to the difficulty in optimizing the accuracy of OVSs, which are also the key parameters of the optical system for achieving reciprocal dual-crystal OVSs. Then, the closed-loop circuit eliminates the DC component of demodulated interference light intensity to suppress the drift of optical parameters with temperature. Considering the fluctuation of *ν* and *θ*, we further propose a control algorithm for improving the detection accuracy of AC/DC voltage. Although our research is based on the dual-crystal scheme to improve the detection accuracy of OVSs, the establishing method of the model of feedback phase considering optical parameters, and the control with exponential *H*_∞_ performance, are still applicable for closed-loop OVSs to suppress the nonlinearity caused by unideal optical parameters. Furthermore, these proposed methods can promote the application of high-precision OVSs in smart grids.

## 4. Experiment

In this section, several experiments are conducted to demonstrate the effectiveness and validity of our analysis. The light source is the super luminescent diode (SLD) with a 1310 nm wavelength. The dual-crystal OVS has adopted the proposed method in this article, and the axis angles are strictly limited in the manufacturing process by the fiber fusion splicer. The dimensions of the BGO crystal in the dual-crystal structure is 5 mm × 5 mm × 10 mm. The voltage measured is provided by a high-precision voltage source and a high-precision transformer. The voltage source adopted in our experiments has an output range of 0–220 V AC and the detection sensitivity of the voltage source is 20 mV. The ratio of the transformer is 220:5000 and the accuracy of the transformer is 0.05 level.

**Figure 7:**
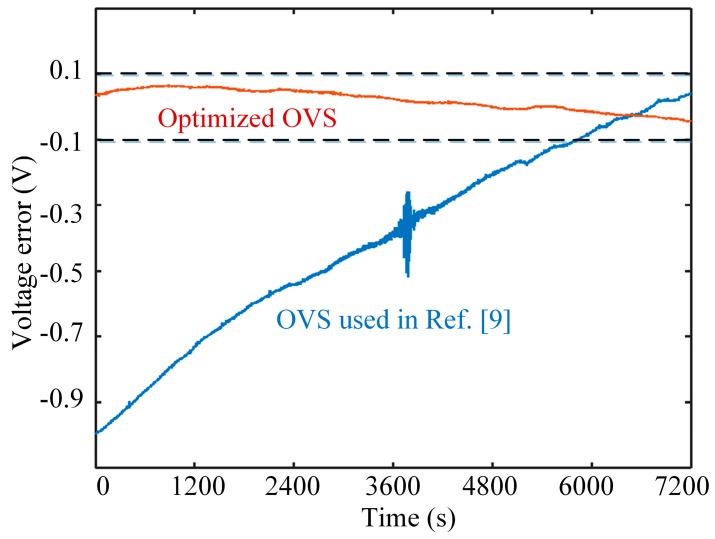
Comparison between the bias stability of the optimized OVS and the previous OVS used in Ref. [9].

Firstly, we conduct the experiment about bias stability of OVSs. In this experiment, the bias stability of OVSs with a dual-crystal scheme is continuously tested for 2 h under zero input, where the bias stability of the dual-crystal OVS adopted in [9] is also tested as a comparison. Figure 7 shows that the additional bias of optimized OVS is less than ±0.1 V under zero input and is greatly reduced, which indicates that the additional bias is smaller due to strict limitation of the axis angles and the suppression effect of control algorithm on the nonlinearity of OVSs.

**Figure 8:**
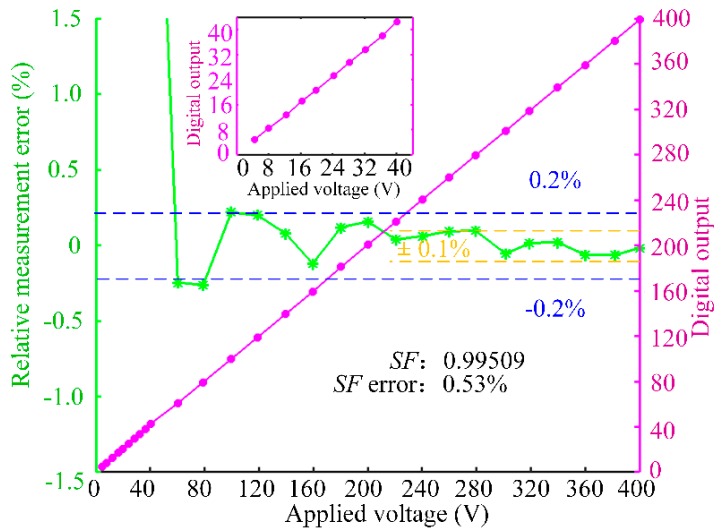
The detection accuracy of OVS with low voltage applied.

Furthermore, we carried out the experiment with small voltage to test and verify the detection sensitivity of OVSs. The sensitivity of OVSs is tested while the applied voltage (0–400 V) is provided by the transformer. The relative measurement error is calculated by ζ = 100% × (*U*_out_/*SF* − *U*)/*U*, where *U*_out_ is the digital output of OVSs. Digital output *U*_out_ is the summation of *u(k)* in 1ms, so that the update rate of output of OVSs is 1 ms. The term *SF* is the scale factor, which means the ratio between the output of OVS and the applied voltage. The error of *SF* is calculated by *ξ* = 100% × (*SF*_max_ − *SF*_min_)/(*SF*_max_ + *SF*_min_), where *SF*_max_ and *SF*_min_ are the maximum and minimum *SF* in the experiment, respectively. The measurement results, as shown in Figure 8, demonstrate that there is good linearity between the digital output of OVS and the applied voltage. Figure 8 proves the good linearity of the OVS in the low voltage range 0–40 V. Moreover, the OVS measurement accuracy is better than ±0.2% if the applied voltage is greater than 100 V, and the measurement accuracy is better than ±0.1% with applied voltage greater than 200 V. The result verifies that the closed-loop OVS has reached the design accuracy of 0.1 level above 200 V. And the measurement error can be 0.1% only if the applied voltages exceed 500 V in [9] (p. 5). This verifies the effectiveness of the control algorithm suppressing the nonlinearity of the unideal optical parameters to improve the detection accuracy of OVSs.

**Figure 9:**
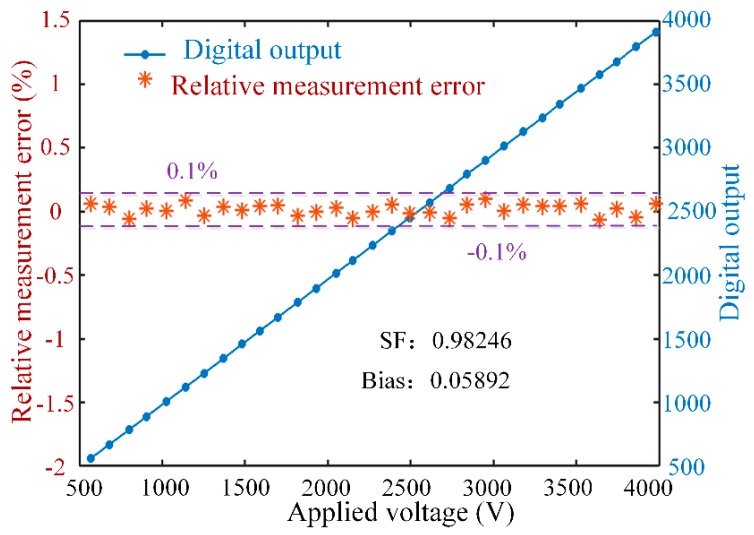
Digital output and relative measurement error of OVSs under voltage 500–4000V.

Finally, the accuracy of OVSs under AC in the voltage range 500–4000 V was measured to further verify the correctness of the control algorithm. The result of digital output and relative measurement error, as shown in Figure 9, indicates that the relative measurement error may be smaller than 0.1% above 500 V AC voltage, which shows the proposed OVS satisfies 0.1 level detection accuracy above 200 V. The *SF* is 0.99509 in the range 0–400 V and 0.98246 in the range 500–4000 V. The error of *SF* is small between the low voltage and the high voltage, which demonstrates the good stability and high detection precision of the OVS. Further, we analyze the detection precision of OVSs under different input voltages in Table 1. This further proves the correctness of the model of feedback phase and the effectiveness of the control algorithm considering the unideal optical parameters.

## 5. Conclusions

The scheme of multi-optical devices enhances the reciprocity of the optical system based on a dual-crystal structure, but the cross-effect of multi-parameters makes it difficult to improve the accuracy of the OVS, especially in practical engineering. In this work, we investigate the quantification model of the feedback phase of a closed-loop OVS, considering the unideal optical parameters. Firstly, the axis angles are limited within a certain range in the manufacturing process so that the influence of axis angles on OVSs is far below the demand of achieving 0.1 level accuracy. Then, we determine the main optical parameters of the detection accuracy of OVSs, which are also the key parameters for achieving reciprocal dual-crystal OVSs. Furthermore, the impact of unideal optical parameters causing the nonlinearity of OVSs is analyzed through simulation results. Furthermore, considering the fluctuation of main unideal optical parameters, we propose a control algorithm to optimize the detection precision of OVSs. The experimental results show that additional bias caused by undesirable optical parameters is obviously decreased and the OVS achieves a measurement accuracy of within ±0.1% for the applied voltage above 200 V, which satisfies the demand of IEC60044-3 for 0.1 level measurement accuracy. This further proves the correctness of the quantification model of feedback phase and the effectiveness of the control algorithm. Moreover, these strategies can promote OVSs to meet the standard for high accuracy of voltage measurement in the application of the smart grid.

## Figures and Tables

**Table 1:** The results of relative measurement error of OVSs.

**Voltage (V)**	40–80	80–200	200–400	>500
**Relative Measurement Error**	>0.2%	0.1–0.2%	<0.1%	<0.1%

## Appendix A

In this appendix, we mainly provide the control algorithm to suppress the unsatisfactory optical parameters in closed-loop OVS.

Based on the previous derivation, we can see that the feedback phase of the system is mainly a function of the Pockels phase 2*δ*, the unideal optical parameters *ν* and *θ*. So we can define a function *f*(2*δ*) = Δ*ϕ_f_*(*t*). It can be seen from the Equation (11) that the uncertain parameters of optical devices lead to the nonlinearity of OVS. We choose 2*δ* as the underlying parameters, then the feedback phase Δ*ϕ_f_*(*t*) can be approximated as

(A1) $$\Delta \varphi_{f}(t)=\frac{-2\cos\theta \cdot \sin2\nu }{\left(\cos4\theta -\cos4\nu \right)}2\delta +\frac{-\cos\theta \cdot \sin2\nu \left(2+\cos4\nu -\cos4\theta \right)}{{\left(\cos4\theta -\cos4\nu \right)}^{2}}{\left(2\delta \right)}^{3}+\ldots$$

Taylor series is used to obtain the relationship between the feedback phase and the unideal optical parameters of the OVS, so that the actual feedback phase is acquired.

In the engineering practice, the actual device parameters are nearly equal to their ideal values, which means *θ* ≈ 180° and *ν* ≈ 45°. Then we conclude that 2 + cos4*ν* − cos4*θ* ≈ 0. Thus, the feedback phase Δ*ϕ_f_*(*t*) can be approximated by

(A2) $$\Delta \varphi_{f}(t)\approx \frac{-2\cos\theta \cdot \sin2\nu }{\left(\cos4\theta -\cos4\nu \right)}2\delta$$

So the nonlinearity factor of the closed-loop OVS is as follows

(A3) $$NF=\frac{-2\cos\theta \cdot \sin2\nu }{\left(\cos4\theta -\cos4\nu \right)}$$

We can note that *f*(2*δ*) is a monotonically increasing and differentiable function for 2δ∈(−π,π). Moreover, *f*(2*δ*) locally satisfies a Lipschitz condition 1 ≤ *f*(2*δ*)/2*δ* ≤ 1.06 with the error of *ν* or *θ* no more than ±5° in practical engineering. That is, there exists a Lipschitz constant *k_l_* ≥ 1.06 such that *f*^T^(2*δ*)(*k_l_*2*δ* − *f*(2*δ*)) ≥ 0 and 2*δ* = *k*_2_*K*_c_*x*(*k*), where *k*_2_*K*_c_*x*(*k*) is the actual feedback signal generated by the controller of closed-loop OVS. Then for any constant *ε* > 0, we can obtain the following condition $\varepsilon f^{T}(k)(k_{l}k_{2}K_{c}x(k)-f(k))\geq 0$.

Select the following Lyapunov-Krasovskii function $V(k)=x^{T}(k)Px(k)$ for system (8) and define $\Gamma (k)=\frac{1}{n}x^{T}(k)x(k)-\gamma^{2}\omega^{T}(k)\omega (k)$. Then, we have that

(A4) $$\begin{array}{l} E\left(V(k+1)-\lambda^{2}V(k)+\Gamma (k)+\lambda^{2}V(k)-\Gamma (k)\right)\\ =E\left(\begin{array}{l} x^{T}(k+1)Px(k+1)-\lambda^{2}x^{T}(k)Px(k)+\Gamma (k)+\lambda^{2}V(k)-\Gamma (k)\\ +\varepsilon f^{T}(k)(k_{l}k_{2}K_{c}x(k)-f(k))) \end{array}\right) \end{array}$$

Let $\eta (k)={\left[x^{T}(k)\;f^{T}(x(k))\;\omega^{T}(k)\right]}^{T}$, then Equation (A4) can be simplified as

(A5) $$\begin{array}{c} E\left(V(k+1)-\lambda^{2}V(k)+\Gamma (k)+\lambda^{2}V(k)-\Gamma (k)\right)\\ =E\left(\eta^{T}(k)[\Theta +\Omega^{T}P\Omega ]\eta (k)+\lambda^{2}V_{m}(k)-\Gamma (k)\right) \end{array}$$

where $\Theta =\left[\begin{array}{ccc} -\lambda^{2}P+\frac{1}{n}I & \varepsilon k_{l}k_{2}K_{c}G & 0\\ 0 & -\varepsilon I & 0\\ \varepsilon k_{l}k_{2}G^{T}K_{c}^{T} & 0 & -\gamma^{2}I \end{array}\right]$. Since the Equation (14) is true, we can obtain that

(A6) $$E\left(V(k+1)\right)<E\left(\lambda^{2}V(k)-\Gamma (k)\right)\leq {\left(\lambda^{2}\right)}^{k}V_{0}(k_{0})-\sum_{s=k_{0}}^{k-1}{\left(\lambda^{2}\right)}^{k-s-1}E\left(\Gamma (s)\right)$$

Furthermore we can obtain that

(A7) $$\sum_{s=k_{0}}^{\infty }E\left(\frac{1}{n}x^{T}(s)x(s)\right)\sum_{k=s+1}^{\infty }{\left(\lambda^{2}\right)}^{k-s-1}<\sum_{s=k_{0}}^{\infty }\omega^{T}(s)\omega (s)\sum_{k=s+1}^{\infty }{\left(\lambda \Phi \right)}^{2(k-s-1)}$$

Finally we conclude that

(A8) $$\sum_{s=k_{0}}^{\infty }E\left(\frac{1}{n}x^{T}(s)x(s)\right)<\gamma^{2}\sum_{s=k_{0}}^{\infty }\omega^{T}(s)\omega (s)$$

Thus, the *H*_∞_ performance can be achieved. This appendix is proof.

## References

[1] Ribeiro B., Werneck M.M., Silva-Neto J.L. (2013). Novel optimization algorithm to demodulate a PZT-FBG sensor in AC high voltage measurements. IEEE Sens. J..

[2] Allil R.C., Werneck M.M. (2011). Optical high-voltage sensor based on fiber Bragg grating and PZT piezoelectric ceramics. IEEE Trans. Instrum. Meas..

[3] Ferrari J.A., Flores J.L., Dultz W., Frins E. (2009). Optical current and voltage sensor using differential spectroscopy. Opt. Eng..

[4] Petricevic S.J., Mihailovic P., Radunovic J. (2009). A miniature Pockels cell with novel electrode geometry. Sensors.

[5] Lee K.S. (1989). New compensation method for bulk optical sensors with multiple birefringences. Appl. Opt..

[6] Lee K.S. (1990). Electro-optic voltage sensor: Birefringence effects and compensation methods. Appl. Opt..

[7] Cecelja F., Balachandran W. (2002). Compensation of environmental effects in bulk optical sensors. IEEE Trans. Instrum. Meas..

[8] Zhang C., Feng X., Liang S., Zhang C., Li C. (2010). Quasi-reciprocal reflective optical voltage sensor based on Pockels effect with digital closed-loop detection technique. Opt. Commum..

[9] Li L., Zhang W., Li H., Pan R. (2013). Linear birefringence-free optical voltage sensor based on dual-crystal structure. Appl. Opt..

[10] Li H., Fu Z., Liu L., Lin Z., Deng W., Feng L. (2017). Analysis of the Light Propagation Model of the Optical Voltage Sensor for Suppressing Unreciprocal Errors. Sensors.

[11] Xiao X., Xu Y., Dong Z. (2015). Thermodynamic modeling and analysis of an optical electric-field sensor. Sensors.

[12] Chu W.S., Kim S.M., Wu X.P., Liu W., Oh M.C. (2016). Optical Voltage Sensors Based on Integrated Optical Polarization-Rotated Reflection Interferometry. J. Lightw. Technol..

[13] Chatrefou D., Montillet G.F. (2003). A series of implementation of optical sensors in high voltage substations. Proc. Transmiss. Distrib. Conf. Expos..

[14] Pan F., Xiao X., Xu Y., Ren S. (2011). An optical AC voltage sensor based on the transverse Pockels effect. Sensors.

[15] Pan F., Xiao X., Xu Y., Ren S. (2012). Optical AC Voltage Sensor Based on Two Bi_4_Ge_3_O_12_ Crystals. IEEE Trans. Instrum. Meas..

[16] Li H., Cui L., Lin Z., Li L., Wang R., Zhang C. (2013). Signal Detection for Optical AC and DC Voltage Sensors Based on Pockels Effect. IEEE Sens. J..

[17] Li H., Bi L., Li L., Hu S., Feng X., Zhang C. (2013). Tracking algorithm for the gain of the phase modulator in closed-loop optical voltage sensors. Opt. Laser Technol..

[18] Shen B., Wang Z., Hung Y. (2010). Distributed *H*_∞_-consensus Filtering in Sensor Networks with Multiple Missing Measurements: The Finite-horizon Case. Automatica.

[19] Song H., Yu L., Zhang W. (2011). Networked *H*_∞_ Filtering for Linear Discrete-time Systems. Inf. Sci..

